# Dynamic Interactions between Tumor Cells and Brain Microvascular Endothelial Cells in Glioblastoma

**DOI:** 10.3390/cancers14133128

**Published:** 2022-06-27

**Authors:** Erika Testa, Claudia Palazzo, Roberta Mastrantonio, Maria Teresa Viscomi

**Affiliations:** 1 Sezione di Istologia ed Embriologia, Dipartimento di Scienze della Vita e Sanità Pubblica, Università Cattolica del Sacro Cuore, L.go F. Vito 1, 00168 Roma, Italy; claudia.palazzo@unicatt.it (C.P.); roberta.mastrantonio@unicatt.it (R.M.); 2 IRCCS, Fondazione Policlinico Universitario “Agostino Gemelli”, L.go A. Gemelli 8, 00168 Roma, Italy

**Keywords:** endothelial cells, cancer, extracellular vesicles, miRNA, angiogenesis, tumor vessels normalization, neovascularization

## Abstract

**Simple Summary:**

In glioblastoma (GBM), tumor cells develop a symbiotic relation with brain microvascular endothelial cells (BMECs) to shift tissue homeostasis toward a tumor-supporting context. Disentangling the molecular mechanisms that govern this dynamic interaction in the context of GBM represents an exciting challenge for the update of conventional treatment and for the development of novel therapeutic targets for this aggressive and lethal brain tumor.

**Abstract:**

GBM is the most aggressive brain tumor among adults. It is characterized by extensive vascularization, and its further growth and recurrence depend on the formation of new blood vessels. In GBM, tumor angiogenesis is a multi-step process involving the proliferation, migration and differentiation of BMECs under the stimulation of specific signals derived from the cancer cells through a wide variety of communication routes. In this review, we discuss the dynamic interaction between BMECs and tumor cells by providing evidence of how tumor cells hijack the BMECs for the formation of new vessels. Tumor cell–BMECs interplay involves multiple routes of communication, including soluble factors, such as chemokines and cytokines, direct cell–cell contact and extracellular vesicles that participate in and fuel this cooperation. We also describe how this interaction is able to modify the BMECs structure, metabolism and physiology in a way that favors tumor growth and invasiveness. Finally, we briefly reviewed the recent advances and the potential future implications of some high-throughput 3D models to better understanding the complexity of BMECs–tumor cell interaction.

## 1. Introduction

Proper brain function requires a highly balanced and monitored extracellular environment where homeostasis is maintained. This highly regulated environment is the result of the functions of the blood–brain barrier (BBB) and the microcirculation of the central nervous system (CNS) [1]. The BBB comprises endothelial cells—the BMECs—connected by tight junctions (TJs) and supported through astrocyte projections with pericytes embedded in the basement membrane. In a healthy BBB, BMECs—in collaboration with astrocytes and pericytes—control the delivery of polar solutes into the CNS through energy-dependent, carrier-mediated systems that transport amino acids, monocarboxylic acids, nucleosides and vitamins. In the case of a brain tumor such as a GBM, the new environment induces modifications of the physical and metabolic properties of the BBB, which is then renamed the blood–brain tumor barrier (BTB) [2]. In the resulting BTB, BMECs lose many of their intrinsic properties and become the main partners of tumor cells by releasing molecules or responding to a signal that helps to monitor the progression of tumor cells [3,4]. Through this partnership, on the one hand, BMECs assist glioblastoma stem cells (GSCs) self-renewal and maintenance by promoting putative stemness characteristics and survival [5,6]. On the other hand, BMECs can stimulate the formation of new blood vessels owing to the GSCs’ proangiogenic capabilities [7,8,9,10,11]. As BMECs and GSCs coexist, this interaction may intervene in defining GSCs’ metabolic plasticity. Therefore, there has been increasing interest in understanding the metabolic and functional plasticity of GSCs and their ability to adapt to the tumor microenvironment (TME) through an intense crosstalk with BMECs [12,13].

This review first summarizes how this interaction modifies BMEC structure, metabolism and physiology favoring tumor growth and invasiveness. We then describe some routes of communication employed in this interaction, and finally, we briefly review the recent advances and the potential implications of some high-throughput 3D models to better understand the complexity of the BMEC–tumor cell interaction.

## 2. Brain Microvasculature in GBM: Shifting from Normal to Tumoral

### 2.1. BMECs in Physiological Condition

BMECs constitute a distinct category of endothelial cells (ECs) for their own features and properties, since they are the interface between the CNS and the blood. First, a distinctive feature of the BMECs is their polarization: they show luminal and abluminal cell membranes differing in their lipid, receptor and transporter compositions. This polarization influences blood–immune system–brain communication since BMECs can respond to different stimuli received from one side of the barrier by releasing cytokines or other molecules [14]. Moreover, BMECs differ from the ECs owing to the presence of specific transporters and receptors that regulate the flux of metabolites across cells, the abundant presence of high-electrical resistance TJs that control the movement between adjacent cells and for low levels of transcytotic vesicles and the lack of fenestrae [15,16,17,18]. However, although BMECs with TJs retain the physical structure of the BBB, the increased complexity and continuity of BMECs are enhanced by astrocytes and pericytes [19]. These cell–cell interactions regulate angiogenesis, microvascular stability, angioarchitecture during CNS development, vascular remodeling [20], and metabolic homeostasis [21].

Recent studies have reported novel BMEC functions. They can actively participate in both innate and adaptive immunity and can amplify the immune response by producing cytokines, prostaglandins and nitric oxide (NO) [22,23,24,25]. In addition, in the so-called oligo-vascular niche [26]—a microenvironment between BMECs and oligodendrocyte— oligodendrocytes and BMECs communicate with each other via the secretion of soluble factors to maintain white matter homeostasis. Some of these factors, such as vascular endothelial growth factor (VEGF) and matrix metalloproteinase-9 (MMP-9), may actively worsen pathological processes (e.g., BBB breakdown), but may promote brain remodeling after injury [27]. Although the mediators of active crosstalk between BMECs and the other cells of the neurovascular unit (NVU) such as neurons, microglia, astrocytes and pericytes are largely unknown, communication is not only achieved through transporters, but also through molecules that are produced and secreted by cells of both the brain and blood interfaces. These secreted substances, such as NO, prostaglandins and cytokines [24,28,29,30,31], can engage autocrine and/or paracrine mechanisms to signal to other cells of the NVU and distal cells in the brain and periphery.

### 2.2. BMECs in BTB

In GBM, the BTB is the consequence of an increased metabolic rate of tumor cells and the upregulation of VEGF, which primes and controls BMECs metabolism and function. Secreted VEGF induces different transformational changes in the brain vasculature—alone or in cooperation with the TME signals—and the growth of structurally altered capillaries from the existing vessels. The BTB is characterized by a reduced expression of tight junctions, altered pericyte coverage, disorganized perivascular astrocyte endfeet and breakdown of the basal membrane, resulting in a heterogeneous increase in vascular permeability (for a review [2]).

Beyond disruptions of the BBB, perturbations of BMECs signaling may affect neuronal function and survival and, more importantly, the proliferation and spreading of tumor cells [32]. The ability of tumor bulk to grow in the surrounding nervous tissue is mainly due to the interplay between a subpopulation of tumor cells, the GSCs, and BMECs. This exchange involves different communication routes that include secreted molecules, gap junctions, tunneling nanotubes and extracellular vesicles, allowing tumor growth and progression [33,34,35,36]. Through this interaction, normal BMECs are hijacked to change their phenotype and function, becoming tumor BMECs. Although the origin of tumor BMECs at present is not well known [37], the microscopic and ultrastructural analyses of GBM-associated microvasculature have contributed to their characterization and differentiation from normal BMECs. First, the phenotypic characterization of GBM-associated blood vessels has shown that they appear as larger and more dilated than their normal counterparts but also tortuous. They show an irregular lumen [38], with an increased number of endothelial caveolae and fenestrations, prominent pinocytotic vesicles, and the lack of perivascular glial endfeet [39]. Furthermore, they are hyperpermeable to plasma proteins, leading to local edema and the extravascular clotting of plasma. The different structural composition of tumoral BMECs leads to abnormal capillary junctions, the formation of aberrant tubules [40] and the opening of the microvessel junctions and subsequent leaking of fluid into the brain. These changes dramatically build and shape the TME and affect various aspects of tumor progression, as the response to therapies.

#### 2.2.1. Characteristics of GBM-Associated BMECs

**Figure 1:**
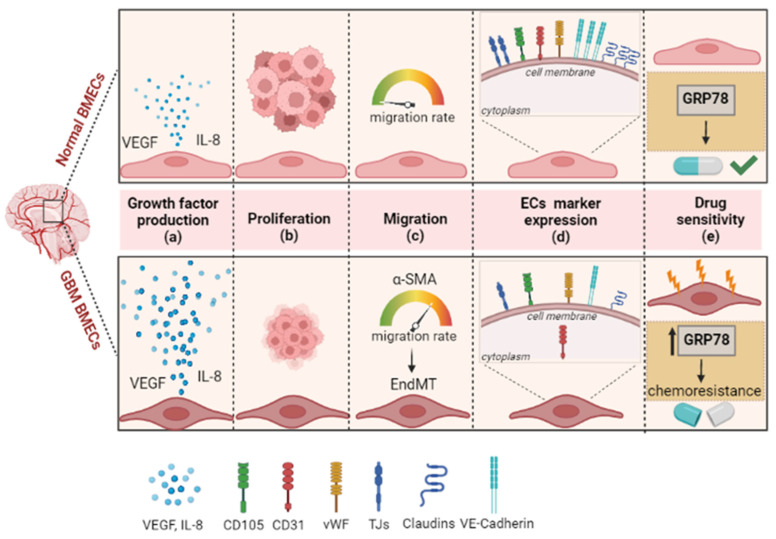
Schematic representation of distinct phenotypic and molecular hallmarks between normal and GBM-associated BMECs. At phenotypic level, GBM-associated BMECs show flat appearance with large nuclei, abundant cytoplasm and veil-like structures (see text for more details). In general, GBM-associated BMECs also change their intrinsic properties by increasing growth factor production such as VEGF and interleukin-8 (IL-8) (blue dots) (**a**); GBM-associated BMECs present a lower proliferation rate than normal BMECs (**b**); the migratory ability is increased in GBM-associated BMECs. The increased expression of some migration factors such as α-SMA and the interaction with brain tumoral cells lead to the endothelial-to-mesenchymal transition (EndMT) process in these cells (**c**); moreover, GBM-associated BMECs show the typical endothelial markers such as vWF and CD105—similarly to normal BMECs— but with a different expression level of VE-cadherin and of TJs and Claudins. More interestingly, the GBM-associated BMECs present some differences in the localization of the CD31, an endothelial cell marker, which is mainly localized into the cytoplasm rather than on the surface membrane (**d**); molecular alterations and the acquisition of intrinsic feature (thunders) lead to the chemoresistance in the GBM-associated BMECs. GRP78 overexpression in GBM-associated BMECs has been shown to confer chemoresistance to several drugs (two-tone pill) used in GBM treatment (**e**).

In the BTB, BMECs display some features that are typical of the primary tumor and that differentiate these cells from healthy BMECs [41,42,43] (Figure 1). First, BMECs present a flat appearance with large nuclei, abundant cytoplasm, multiple nucleoli and veil-like structures [44,45], losing their canonical cobblestone-like appearance [37]. At the molecular level, although GBM-associated BMECs present typical endothelial markers, such as vWF, CD105, CD31 and VE-cadherin [46,47,48,49], their expression level is quite different. In particular, it has been shown that GBM-associated BMECs present a lower expression of VE-cadherin (CD144)—a TJ protein that plays an important role in the integrity of the BBB—as well as other TJs proteins, such as claudin-1, claudin-5 and occludin, compared to normal BMECs [50,51]. Moreover, they show a different subcellular localization of the CD31 protein—also known as platelet endothelial cell adhesion molecule-1—as it is distributed more in the cytoplasm than in the surface membrane [37]. Furthermore, at least 50% of GBM-associated BMECs express α-SMA, a cytoskeletal protein directly related to cell migration and commonly expressed by pericytes [37]. In addition, they exhibit different proliferative and growth properties compared with normal BMECs. Indeed, they migrate faster than normal BMECs, but, more interestingly, their migration process is not based on chemotaxis, but on chemokinetic, indicating that GBM-associated BMECs are active in the absence of exogenous factors [37] (Figure 1). This suggests the possibility that de novo expression of markers such as α-SMA, or others, may enhance the migratory ability of these cells and the progressive acquisition of phenotypical and functional characteristics of mesenchymal cells [45]. In this endothelial-to-mesenchymal transition of the BMECs the collaboration between tumor growth factor-β (TGF-β) and Notch pathways plays a crucial role [52]. Another typical property of the GBM-associated BMECs is that they show a lower proliferation rate than normal brain BMECs [37], although they exhibit a downregulation of pro-apoptotic genes and an upregulation of anti-apoptotic genes [53]. More interestingly, this feature may be among the causes of the intrinsic resistance of these cells to chemotherapeutic agents, which has also been associated with the reduction of GRP78 protein [54].

#### 2.2.2. Key Molecular Features of GBM-Associated BMECs

Studies on patients and animal models of GBM have revealed the specific molecular alteration of BMECs [2], providing crucial information for the intra-tumoral distribution of these cells. Through the analysis of bulk mRNA isolated from BMECs, GBM-associated BMECs are associated with a distinct gene signature [55,56]. The development of a single-cell transcriptome strategy of freshly isolated BMECs from human GBM provided the first scRNA-seq-based molecular atlas of the human BMECs [57]. This elegant study of Xie and colleagues has characterized different BMEC clusters, each associated with distinct anatomical localizations and molecular phenotypes. While the BMECs in the periphery of the tumor displayed a quiescent endothelial marker profile characterized by a high expression level of genes implicated in vascular integrity, the BMECs of the tumor core showed an endothelial angiogenic phenotype and a gene signature associated with vascular basement membrane remodeling, cytoskeletal rearrangements and angiogenic sprouting. Furthermore, BMECs in the tumor core displayed the upregulation of genes associated with metabolic pathways, including glycolysis, the citrate cycle, oxidative phosphorylation, nucleotide synthesis and the downregulation of genes associated with glutamate metabolism, suggesting that the high glycolysis in tumor BMECs is a mirror of the tumor context, and reflects the high demand for energy in angiogenesis.

These exciting new data mark the beginning of a deeper understanding of the characteristics and functional properties of BMECs, as well as their spatial alteration in GBM. Moreover, they may provide key information about the intra-tumoral distribution of BMECs in GBM and advance the design of customized therapeutic treatments and drug delivery to halt tumor growth.

## 3. Metabolic Interactions between Tumor Cells and BMECs in GBM

In GBM, as in other cancers, tumor cells respond and adapt to tissue changes and the biochemical context [58,59]. GBM arises in a hypoxic environment; thus, GBM cells are forced to adapt to hypoxia by shifting their behavior, which results in genetic, epigenetic, post-transcriptional and metabolic changes [60]. To survive and proliferate, GBM cells use multiple catabolic pathways for energy production. For example, they use glycolysis, which both supports energy production and enables tumor cells to use glucose-derived carbons for the synthesis of nucleic acids. Furthermore, they can use other sources of energy, such as amino acids and nucleotides, which are stored through a variety of molecular mechanisms, including extracellular uptake, de novo synthesis, fluxing carbons and nitrogens through a variety of different bioenergetic pathways [59]. Nutrients derived from the microenvironment also regulate signaling pathways through nutrient sensors within GBM cells, such as mTORC1 and AMP-activated protein kinase (AMPK), supporting the bioenergetic demands of the cells and thus critically contributing to tumor biology [58].

Studies on metabolic pathways in cancer have mainly focused on understanding the similarities and differences in metabolism between ECs and cancer cells. In this field, while the metabolic features of the tumor cells are dictated by their intrinsic needs, ECs adapt their metabolism to generate additional energy in order to meet the demands of the tumor cells. The metabolic adaptation of BMECs during angiogenesis is well-documented [61]. For several crucial metabolic pathways, it seems that BMECs may resemble cancer cells, but available information is inadequate to draw definitive conclusions on the topic. More importantly, as cancer cells, BMECs also become highly glycolytic [62]. This switch seems counterintuitive, since BMECs are in contact with blood and, therefore, with a direct and unlimited source of oxygen and glucose, suggesting an ideal environment for oxidative phosphorylation, it may present several advantages. First, a highly glycolytic environment allows both cell types to proliferate in the hypoxic tumor context. Then, by using glycolysis, BMECs could hypothetically protect themselves and perivascular cells from oxidative stress, allowing them to survive and meet the energy request of tumor cells. Moreover, since glycolysis seems to be an important regulator of angiogenesis that is closely intertwined with angiogenic signals, it may explain the high metabolic demand necessary for the migration and proliferation of BMECs during angiogenesis [63]. However, as BMECs and cancer cells exhibit differences in their metabolic needs, pathways and mechanisms, additional studies are needed to understand the dynamics of the BMEC metabolism, which should provide interesting avenues for therapeutic strategies to block tumor growth.

## 4. Signaling Molecules Participating in BMECs-Tumor Cells Communication

**Figure 2:**
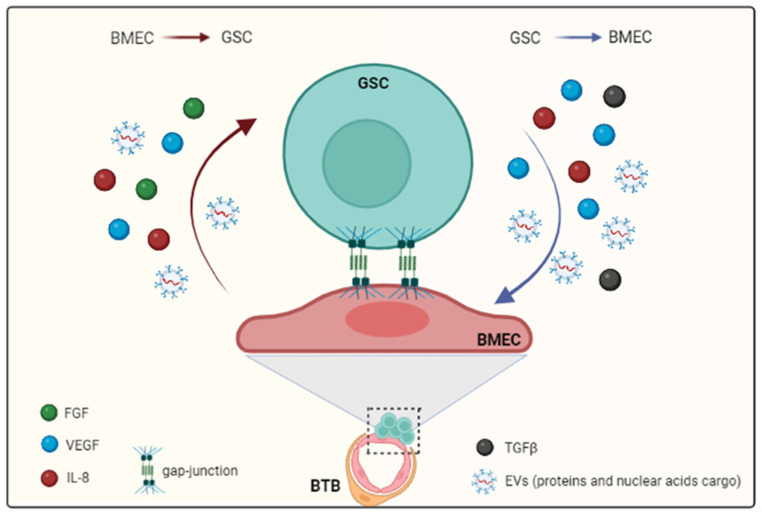
GSCs and BMECs communication routes. GBM induces modifications of the physical and metabolic properties of the BBB, becoming a BTB. In the resulting BTB, BMECs become the main partners of GSCs and their communication follows dynamic and bi-directional routes. This interaction occurs by direct cell contact (gap junction) or by paracrine signaling. The secreted effector molecules are growth factors (VEGF, FGF and TGF-β) and cytokines such as IL-8. In addition, EVs, in which proteins and nuclear acids are the main cargo, are an alternative route of communication (see text for more details).

Tumor cells develop a symbiotic relation with BMECs to shift tissue homeostasis toward a tumor-supporting context. GSCs–BMECs communication is dynamic and bi-directional and makes use of different routes, including cell–cell transversing gap junctions and the secretion of effector molecules such as growth factors, cytokines, chemokines and extracellular vesicles (EVs) [64,65,66], (Figure 2). The main functions and features of the most prominent proangiogenic factors, such as VEGF, fibroblast growth factor (FGF) and IL-8, are briefly discussed below.

### 4.1. VEGF

VEGF is a potent inducer of angiogenesis [67], and in brain tumors it is both cancer and BMEC derived. VEGF acts via a paracrine and autocrine mechanism [68,69], and it is associated with tumor progression, increased vessel density, invasiveness, metastasis and tumor recurrence. This factor is an important regulator of the endothelial response to changes in metabolic substrate availability [70], through high-affinity binding to the tyrosine kinase receptors VEGFR1 and VEGFR2 [71]. During pathological angiogenesis, VEGF secreted by tumor cells may induce ECs proliferation and survival primarily via the ERK and PI3K/Akt pathways [72], as well as cell migration via multiple signaling pathways, mainly involving the PI3K stimulation and activation of Rho GTPases [73]. Furthermore, VEGF-mediated cell invasion is promoted by the expression of MMP-2, MMP-9 and urokinase plasminogen activator, which degrade the basal membrane and extracellular matrix (ECM), allowing the migration of ECs and the formation of vascular sprouting [74]. Vascular permeability induced by VEGF can be driven by several mechanisms, including junctional remodeling, and the induction of fenestrae and vesiculo-vacuolar organelles [75], a dysregulation mechanism that leads to vascular hyper-permeability to facilitate metastases [76]. Traditionally, brain tumor cells produce VEGF that act upon the ECs via VEGFRs [77,78]. However, it is well-known that tumor-derived VEGF provides not only paracrine survival cues for BMECs, but may also fuel autocrine processes in tumor cells, further complicating the TME. In this regard, it is well-known that other important pro-angiogenic factors, such as neuropilin-1, interact with, and stabilize, VEGFR2 in the presence of VEGF ligand [79]. The VEGF–VEGFR2–NRP1-mediated signaling in GSCs is maintained in an autocrine manner via the continuous secretion of VEGF, allowing for the persistent activation of downstream intracellular pro-survival pathways and promoting tumor growth and resistance to some treatments [79].

VEGF can also be transported from the GBM cells to the BMECs through channel-dependent mechanisms. Direct cell-to-cell communication via Cx43 gap junctions is implicated in the transportation of VEGF from the GBM cells to the BMECs and in promoting tube formation in the latter vessels’ structure and functions [80]. Beyond these conventional methods of VEGF release, it is well established that VEGF can also be embedded in EVs in order to reach cells to bind to its receptors and exert its functions.

**Figure 3:**
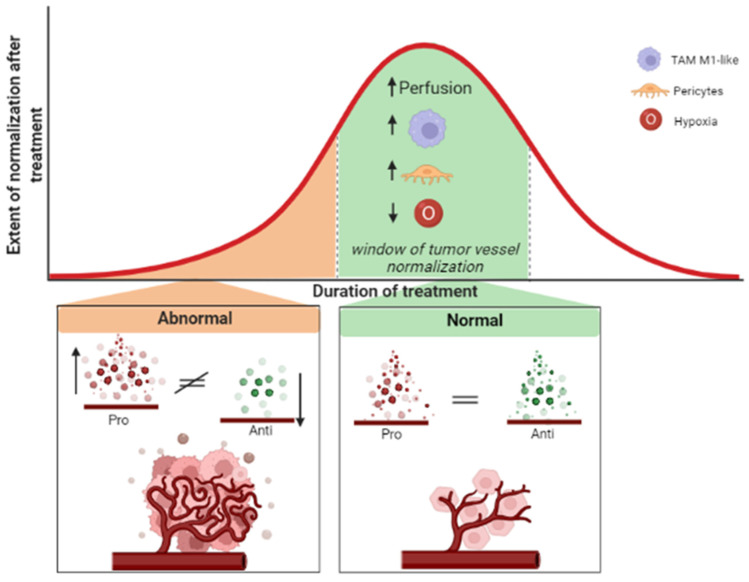
The tumor vessel normalization is one of the main mechanisms of action that drives the use of antiangiogenic therapies. Due to its extensive vascularization, treatment protocols of GBM, in addition to chemotherapeutic drugs, adopt antiangiogenic compounds. However, the efficacy of antiangiogenic compounds on tumor vessels normalization seems dose- and duration-dependent. At the initial stage of the treatment, abnormal tumor vessels are a hallmark of GBM. Then, in the so-called “window of tumor vessel normalization”, through the balance between the pro- and antiangiogenic agents, the process of vessel normalization occurs. Here, the tumor vessels become normal in structure and function; the coverage of blood vessel by pericytes increases and the immune cells shift towards a tumor-associated macrophages (TAM) M1-like, leading to improved vessel perfusion and reduced tissue hypoxia. Nevertheless, the process of vessel normalization is transient and hard to capture: it occurs very quickly and lasts a short time spanning.

Compounds targeting VEGF-mediated pathological angiogenesis have marked the beginning of a new era in GBM treatment [81]. Antiangiogenic treatment is considered a primary approach to tumor vessel normalization that acts through the balance of pro- and antiangiogenic agents and the timing administration of antiangiogenic compounds (for a review: [82]) (Figure 3). Nevertheless, the process of vessel normalization is transient and difficult to capture; it occurs very quickly and does not last long.

### 4.2. FGF

Proteins from the FGF family are involved in various biological functions, such as proliferation, differentiation, migration and angiogenesis (for a review: [83]). Among them, the most recognized proteins are FGF1 and FGF2, also termed acidic fibroblast growth factor (aFGF), and basic fibroblast growth factor (bFGF), respectively, which are potent angiogenic factors. FGFs bind to four high-affinity tyrosine kinase receptors (FGFRs 1–4), of which FGFR1 and -2 may be expressed on the surface of ECs [84]. The FGFs binding to their specific receptors and the subsequent activation of signal transduction cascades induce a strong angiogenic responses on the ECs through both autocrine and paracrine mechanisms [85,86]. While an unspecified role of FGF1 in the proliferation and differentiation of all cell types necessary for building an arterial vessel has been shown, FGF2 seems to be mainly involved in the proliferation of ECs and their organization into tube-like structures [87]. More interestingly, FGF may act as a single proangiogenic factor or in crosstalk with VEGF [87,88]. To this end, FGF signaling is required for the maintenance of the VEGFR2 expression of ECs, as well as their ability to respond to VEGF stimulation [89].

### 4.3. IL-8

IL-8 is among the cytokines that have been extensively studied with a role in directing angiogenesis, invasion and GSC behavior [90]. The detection of an elevated IL-8 concentration at the tumor resection margin and a lower level in the peritumoral region has been one of the drivers leading to the association of this chemokine with invasion and angiogenesis [91], as well as with GBM progression and poor prognosis [92,93,94].

IL-8 is a chemokine with pro-inflammatory properties, whose biological effects are mediated by two receptors: CXCR1 and CXCR2. Both are members of the seven transmembrane G-protein-coupled receptor super families and bind IL-8 with high affinity, even if they can also bind other CXC chemokines [95]. Although in physiological conditions, its expression in the brain is very low, in GBM this chemokine is expressed by many tumor cells and cells of the TME [90]. The presence of IL-8 and its receptors in GBM cells, both in tumor specimens and GBM cell lines, demonstrates both autocrine and paracrine signaling, promoting GBM growth [92,93,96,97,98,99]. IL-8 may act through a paracrine mechanism by upregulating stem cell marker expression in GSCs and activating various signaling pathways associated with tumorigenesis, such as STAT3, PI3K and MAPK [90,93,98,99].

A distinct feature of GBM is its leaky endothelial barrier, which contributes to angiogenesis and edema. Regarding this, it has been shown that BMECs cultured with conditioned medium from GBM cells presented increased permeability due to the remodeling of VE-cadherin, as a result of IL-8-CXCR2 activation [100]. More recently, Guequen and colleagues determined that IL-8 released by GBM cells through the S-nitrosylation of VE-cadherin and p120 can destabilize the endothelial barrier [101].

These studies, together with recent evidence showing that the CXCR2-CXCL2-IL8 signaling has a similar effect on BMECs to VEGF/VEGFR [88], suggest that the inhibition of IL-8 may be an effective way to control and/or block damage to the endothelial barrier and prevent cancer progression.

## 5. EVs as a Novel Unconventional Mechanism of Communication between Tumor Cells and BMECs

In both physiological and pathological conditions, cells use EVs as an additional mechanism of intercellular communication for sharing both signals and supplies [102,103]. EVs are secretory membrane-bound submicron vesicles that can be classified into two broad classes based on their size: exosomes (from 30 nm to 150 nm) and microvesicles (up to 1000 nm), also including apoptotic bodies and oncosomes, which are the largest known vesicles. Beyond size, they differ from each other for biogenesis and composition [104]. Generally, it is reported that EVs encapsulate and transfer molecules including lipids, proteins and nucleic acids, amongst other bioactive materials into the surrounding milieu. The transfer of such materials between cells in the TME, as well as tumor cells, has been shown to facilitate several tumor-promoting mechanisms, including angiogenesis, invasion and metastasis [105]. Therefore, in this review, we aimed to summarize the role of EVs as a novel and additional BMECs–GSCs communication route [106,107]. GBM cells release EVs carrying many pro-angiogenic factors shaping tumor vasculature, including TGF-β, VEGF, proteolytic enzymes, ribonucleases (such as plasminogen activators and angiogenin) and chemokines [108,109,110]. In turn, EVs released or shed by donor cells are able to reprogram the epigenome and transcriptome profile of BMECs, contributing to angiogenesis at the site of release or at a distance from the source of secretion [111,112]. Furthermore, VEGF-A of hypoxic GBM EVs increases BBB permeability in both in vitro and in vivo models by reshaping the expression and organization of claudin-5 and occludin [113,114]. Another property of these vesicles is that they can reach the bloodstream and disseminate at a distance from the primary tumor site progression [109]. More recently, Wang and colleagues [115] found a specific 120 kDa isoform of VEGF, the VEGF-C, in GBM-derived exosomes. By binding to VEGFR2, the VEGF-C showed a strong stimulatory effect on tafazzin expression in BMECs by inhibiting the Hippo signaling pathway, which contributes to the stimulation of EC viability, migration and tubule-like formation.

Although the role of pro-angiogenic factors harbored by EVs in targeting BMECs and their ability to form new vessels have been well documented [109,115,116], the significance of this alternative delivery route is still obscure. The recent evidence that VEGF packaged in EVs derived from breast cancer cells, while triggering the activation of VEGFR2 on ECs, also makes them insensitive to antiangiogenic therapies [117], has led to the hypothesis that EVs might be used in a cunning strategy to allow proangiogenic factors to evade decoy receptors and proteases. These findings are extremely interesting and might explain the ineffectiveness of this VEGF antibody in cancer treatment and help in the design of effective therapeutic treatments.

Another exosome-protein cargo involved in angiogenesis is represented by semaphorin3A (Sema3A). In in vitro studies, this protein, which is exposed on the surface of EVs, is capable of disrupting the endothelial barrier integrity via binding to the neuropilin 1 receptor [105]. In addition, Sema3A carried by EVs derived from the blood of GBM patients induced a significant vascular leakage, a condition that was not observed with EVs derived from healthy volunteers.

Most evidence from the study of the intercellular communication between ECs and glioma cells via EVs has focused on EVs derived from GBM cells and acting on ECs. Very few studies exist on the effect of EC-derived EVs on GSCs. A recent study of Shi and colleagues shed light on the content of BMEC-derived EVs represented by the abundant tetraspanin CD9, which is defined as a GSC biomarker and is able, via STAT3 activation, to significantly increase both GSC proliferation and tumor-sphere formation in vitro and tumorigenicity in vivo. On the other hand, CD9 carried by BMEC-derived EVs seems to inhibit glioma cell growth in vitro, in contrast to the effect on GSCs, but the reason for this inverse biological effect remains unknown [118].

Beyond protein cargo, genetic material carried by EVs contributes to the regulation of GBM angiogenesis. An active player in this field is miR-21, whose levels are significantly elevated in GBM [119]. Exosomal miR-21 derived from GSCs was demonstrated to promote the angiogenic ability of BMECs by stimulating the VEGF/VEGFR2 signaling pathway [110]. To endorse these findings, other studies have shown that promotion of the neo-angiogenesis process can be mediated by several miRNAs packaged in exosomes derived from GSCs, such as miR-26 and miR-9. Through increasing VEGF levels, miR-9 was reported to support tube formation in human BMECs by triggering the PI3K/AKT pathway and enhancing the angiogenic properties of BMECs and tumor growth in nude mice [120]. Moreover, the expression level of miR-9 delivered by GBM-derived exosomes was also correlated with a tube-like-structure formation in HUVEC cells. In both in vitro and in vivo studies, miR-9 also affected the downregulation of three targets (RGS5, SOX7 and ABCB1) implicated in anti-angiogenic pathways in recipient BMECs [121]. In addition, an overwhelmed GBM hypoxic environment generated more EVs compared to normoxic parental cells enriched in hypoxia-regulated mRNAs and proteins. These vesicles, with different cargo compositions, mediate the communication between GBM and BMECs, leading to a strong activation of tumor neovascularization [116]. For example, upon hypoxic stress, several microRNAs were shown to be upregulated, including miR-210, miR-1275, miR-376c, miR-23b, miR-193a and miR-145. Among these, miR-210 is the most upregulated microRNA and can be secreted from GBM cells through EVs, directly affecting BMEC response [122] and repressing glycerol-3-phosphate dehydrogenase 1-like and HIF3A, which stabilizes HIF1A, causing an elevated level of its downstream target VEGF [123]. However, the mechanism by which hypoxia affects tumor angiogenesis via exosomes derived from tumor cells remains largely unknown.

Long non-coding RNAs (lncRNAs) are non-protein coding transcripts that regulate gene expression at epigenetic transcriptional and post-transcriptional levels [122]. They can be transferred by EVs such as miRNA. Increasing evidence has established that the aberrant expression of a subtype of lncRNAs, long intergenic non-coding RNAs (lincRNAs), plays a critical role in tumor biology [123,124]. The EV-based delivery of long non-coding RNA CCAT2 (linc-CCAT2) to BMECs was found to trigger angiogenesis in the GBM both in vitro and in vivo. Indeed, it was reported that lincCCAT2-overexpressed EVs derived from the U87 cell line enhance the EC expression of VEGF-A and TGF-β and alleviate apoptosis via activating B-cell lymphoma-2 (Bcl-2) and inhibiting Bcl2-associated protein x (Bax) and caspase-3 cleavage [125]. Moreover, long non-coding RNA HOX antisense intergenic RNA was shown to induce the expression of VEGF-A in GBM cells promoting neo-angiogenesis in vitro. More interestingly, it exerts this proangiogenic function only when it is encapsulated and delivered via EVs from tumor cells to BMECs [126]. Lastly, very few studies have focused their attention on the emerging role of exosome-mediated metabolic reprogramming in the regulation of TME and cancer progression [113,127]. By performing a proteomic analysis of EVs isolated from the conditioned medium of five GBM cell lines, Naryzhny and colleagues identified a list of 133 proteins, including those involved in the metabolic process. The set of enzymes contained in the exosomes of GBM cells closely mirrored the metabolism of cancer cells, suggesting that the metabolic contents of EVs may also favor glycolytic pathways, making EV internalization an energetically favorable event for the target cells. Understanding the spatiotemporal sequence of metabolic changes in the GBM environment and the role of EVs in these processes can provide a greater understanding of the tumor biology and offer other potential therapeutic targets [128].

## 6. Novel Technical Approaches in the Study of GBM

Although today the gold standard for studying GBM remains the animal model, a vast set of other tools has been proposed [129,130]. In this field, 3D platforms have proven to be a suitable model to gain insight into GBM biology and to disentangle the cell-to-cell interactions in a more physiological environment [129,130].

In 3D tumor platforms, the patient’s tissue-derived cells are co-cultured with different cell populations in a gel-embedded system in order to mimic the complex TME, and then incubated in a classical growth medium [131,132]. In a different setting, cancer cells can be assembled within microfluidic devices, offering the great advantage of working with a reduced number of patient-derived cells. Furthermore, in mechanically supported 3D models, cells are layered in a solid scaffold made up of biomaterials with different mechanical properties and then maintained in a classical growth medium [131,132]. All of these models present different ECM components and different cell types in co-culture and are suitable for studying the interplay and crosstalk among tumor cells and TME cells.

High-throughput 3D models have the potential to fill the gap between the 2D in vitro and in vivo models [132]. Along with the benefits of their low cost and high reproducibility, they overcome several limitations of both the classical 2D cell cultures and in vivo models. In particular, their complex organization makes them more informative than the 2D models, as they recapitulate the GBM milieu without its intrinsic limitations, such as the differences in protein and gene expressions observed in 2D models [133,134,135]. On the other hand, unlike the in vivo models, they are less expensive, less variable and, more importantly, they do not raise ethical problems.

Although several 3D models have had great success in interrogating tumor responses to the TME (for a review: [131,132]), mainly regarding ECM composition, organization, and drug resistance and diffusion, only a few studies have investigated tumor cell–BMEC dynamics. In this regard, more recently, 3D vascularized tumoroid in vitro models confirmed their validity in recapitulating the complex GBM milieu [136,137]. These platforms, although limited by their lack of perfusable vasculature, were also effective in demonstrating the capacity of patient-derived primary GBM cells for sustaining angiogenic sprouting [136] as well as the role of ECs in promoting GBM growth and invasion through IL-8 signaling [137].

Recently, the need to bring these models closer to the biological GBM setting has led to the rapid development of 3D bioprinting models, in which the interaction between tumor cells and the ECs is investigated in microvascular-like structures obtained by positioning ECs within the 3D structures [138,139]. There are currently few examples of the application of this approach to GBM, and they can pave the way for future studies in this field. The recent advances in time-lapse microscopy have gradually led to the switch from 3D models to 4D culture models [140], where it is possible to monitor the dynamic responses through the use of stimuli-responsive biomaterials. 4D models can be employed in the development of high-throughput vascularized GBM models and the testing of anti-tumor drugs, especially neovascularization inhibitors, in a more physiologically relevant setting, accelerating their clinical translation over time.

## 7. Conclusions

The GBM landscape is incredibly complex, and despite all of the advances, additional studies are needed to fully decipher the interactions between the various cell populations of TME and the tumor cells, as well as their specific signaling pathways. In this context, investigating the cellular and molecular mechanisms governing the interactions between BMECs and tumor cells is crucial and can unveil new therapeutic targets for the development of successful and long-lasting anti-GBM treatments. Additional studies are needed to sound other potential routes and factors involved in tumor cells-BMECs communications and, in particular, to characterize their role in the BMECs metabolic switch during the process of neovascularization. Moreover, a better understanding of the contribution of pericytes and astrocytes to the process of neovascularization is essential and may provide novel and relevant therapeutic target for vasculature normalization. Notably, the use of preclinical models such as the 3D BBB bioprinting platforms are considered an opportunity to better understand the role of different cell populations in the complex process of neovascularization. 3D models can include patient BMECs, pericytes, and astrocytes and mimic native GBM features, holding both the potential to identify novel therapeutic targets and to test anti-GBM drugs in a more physiological setting, facilitating their clinical translation.
